# Pressure Properties of a New Positive Expiratory Pressure Device—OpenUp Flow a Three‐in‐One Solution

**DOI:** 10.1111/crj.70084

**Published:** 2025-05-13

**Authors:** Pär Wennberg, Bengt Sundberg, Elisabeth Westerdahl

**Affiliations:** ^1^ School of Health Sciences Jönköping University Jönköping Sweden; ^2^ Faculty of Medicine and Health, University Health Care Research Center Örebro University Örebro Sweden

**Keywords:** breathing exercises, chronic obstructive, pulmonary disease, respiratory therapy

## Abstract

**Introduction:**

A new flow‐regulated PEP device has been evaluated regarding functionality and pressure properties.

**Methods:**

The three different resistance levels were assessed and evaluated at standardized flow rates of 10 and 18 L/min; Kruskal–Wallis test was used to analyse the differences in generated pressure between the different resistance levels.

**Results:**

A range of 3–31 cmH_2_O was generated with airflows of 10 and 18 L/min. There was a significant difference in pressure among different resistance levels at both flow rates.

**Conclusion:**

Overall, there was a significant difference in pressure among different resistance levels at both flow rates, showing that the high resistance significantly increased pressure compared with low resistance. This new device is performing comparable with other resistors available in the market.

## Graphical abstract

A new flow‐regulated PEP‐device has been evaluated regarding functionality and pressure properties at standardized flow rates of 10 and 18 L/min. There was a significant difference in pressure at high resistance significantly increased pressure compared with low resistance levels at both flow rates. This new device is performing comparable to other resistors available in the market.

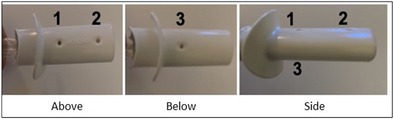

## 1 Introduction

Positive expiratory pressure (PEP) technique is often recommended to patients with chronic obstructive pulmonary disease (COPD) or patients undergoing major surgery [1, 2]. Common indications for PEP breathing include, but are not limited to, increasing lung volume, reducing hyperinflation and improving airway mucus clearance, and it is often combined with other physical therapy techniques [1, 3, 4]. Many of the existing PEP devices on the market are constructed from numerous small pieces, making assembly demanding for patients and can challenge proper cleaning of the device. A flow‐regulated PEP device; OpenUp Flow (Parx AB, Saltsjöbaden, Sweden), has been developed in collaboration with healthcare professionals experienced in treating patients with COPD. The design has focused on ease of use, while meeting hygienic and environmental standards. Evaluation of its functionality and pressure properties is necessary as it enters the market. Hence, the objective of this trial was to assess OpenUp Flow in terms of pressure regulation using standardized flow rates.

## 2 Methods

**FIGURE 1:**
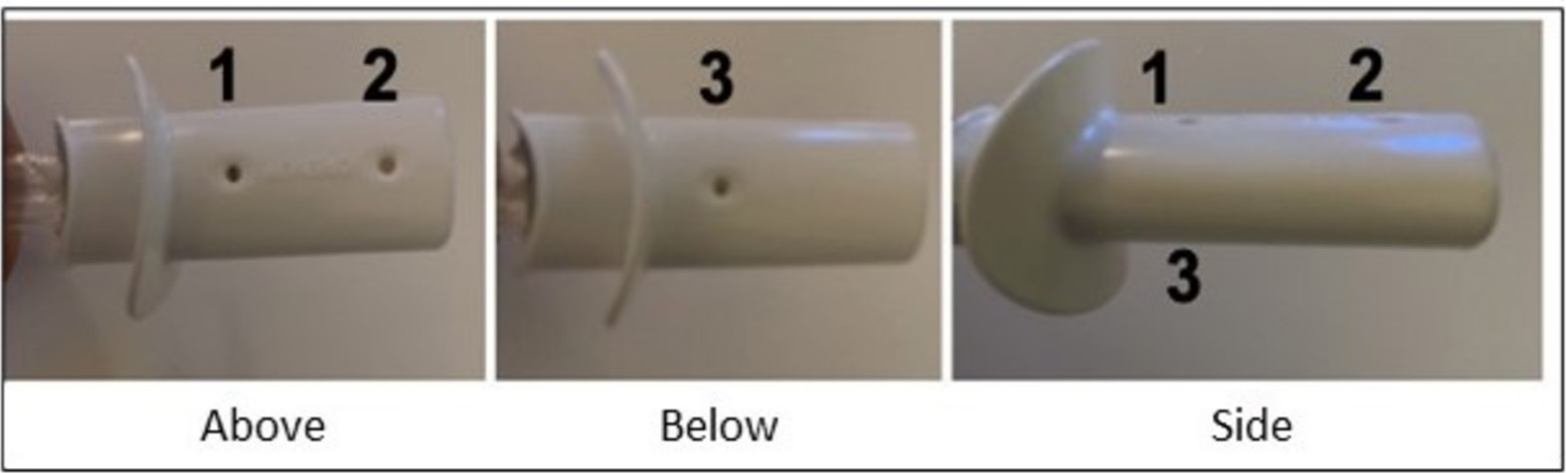
Overview of the different openings of the device OpenUp Flow (Parx AB, Saltsjöbaden, Sweden). The three holes are identified by numbers in the figure.

The PEP device OpenUp Flow comprises an open mouthpiece with a lip‐supportive shield on one end and a blocked opposite end. It is constructed from a single piece of polypropylene to eliminate assembly requirements. The device measures 8.2 cm in length with three standardized 5 mm^2^ openings (holes) (Data S1). The hole diameter and spacing affects flow characteristics. OpenUp Flow is easily cleaned with tap or boiled water and can endure autoclave sterilization up to 134°C in medical settings. Three resistance levels can be established by blocking openings: high resistance (two blocked openings), medium resistance (one blocked opening) and low resistance (no blocked openings) (Figure 1).

**FIGURE 2:**
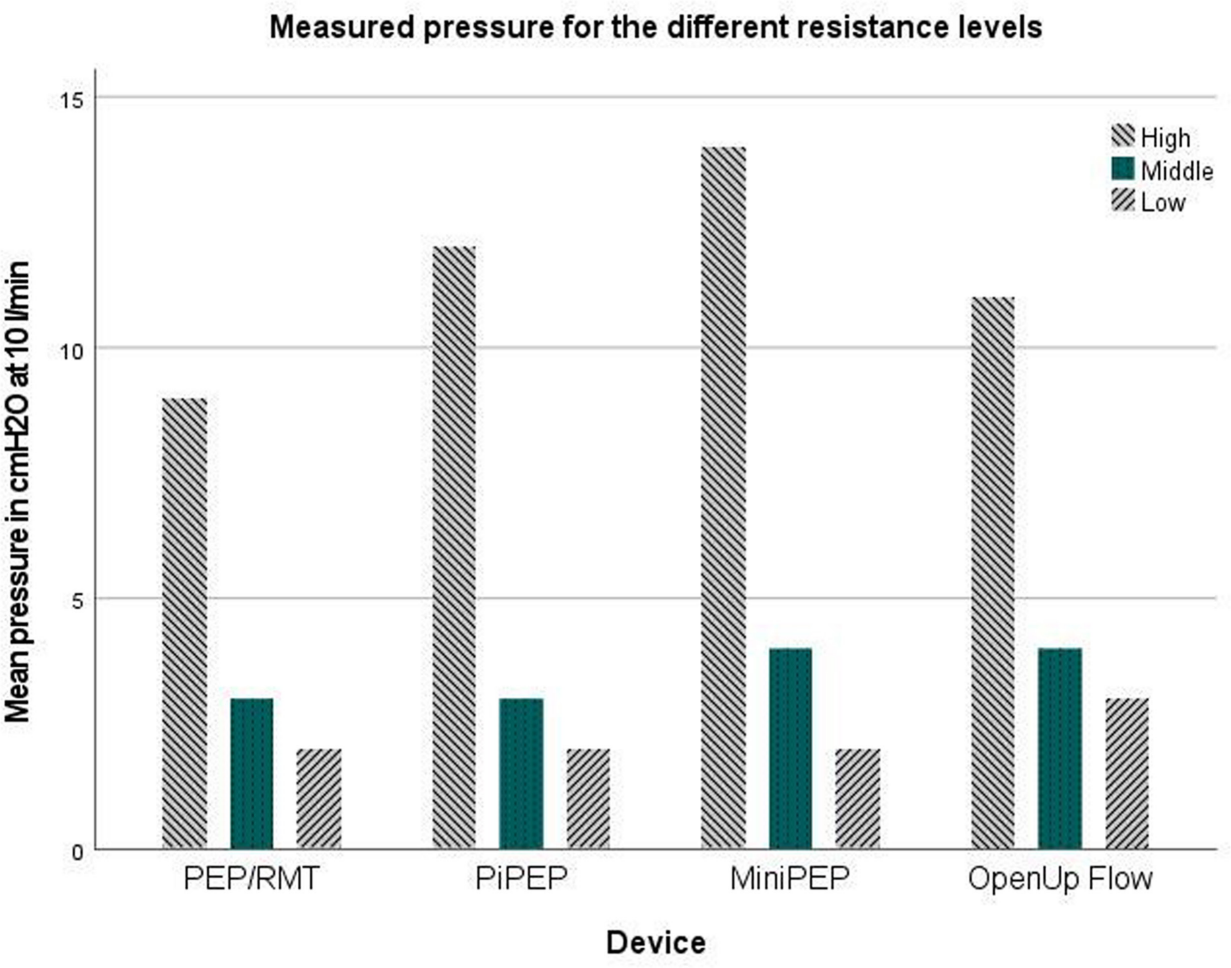
Pressures measured at a flow of 10 L/min in OpenUp Flow, compared with the competitors PEP/RMT system, PiPEP, and MiniPEP. High resistance was compared with 2.0 mm resistors. Middle resistance was compared with 3.0 mm resistors. The low resistance was compared with 3.5 mm resistors.

**FIGURE 3:**
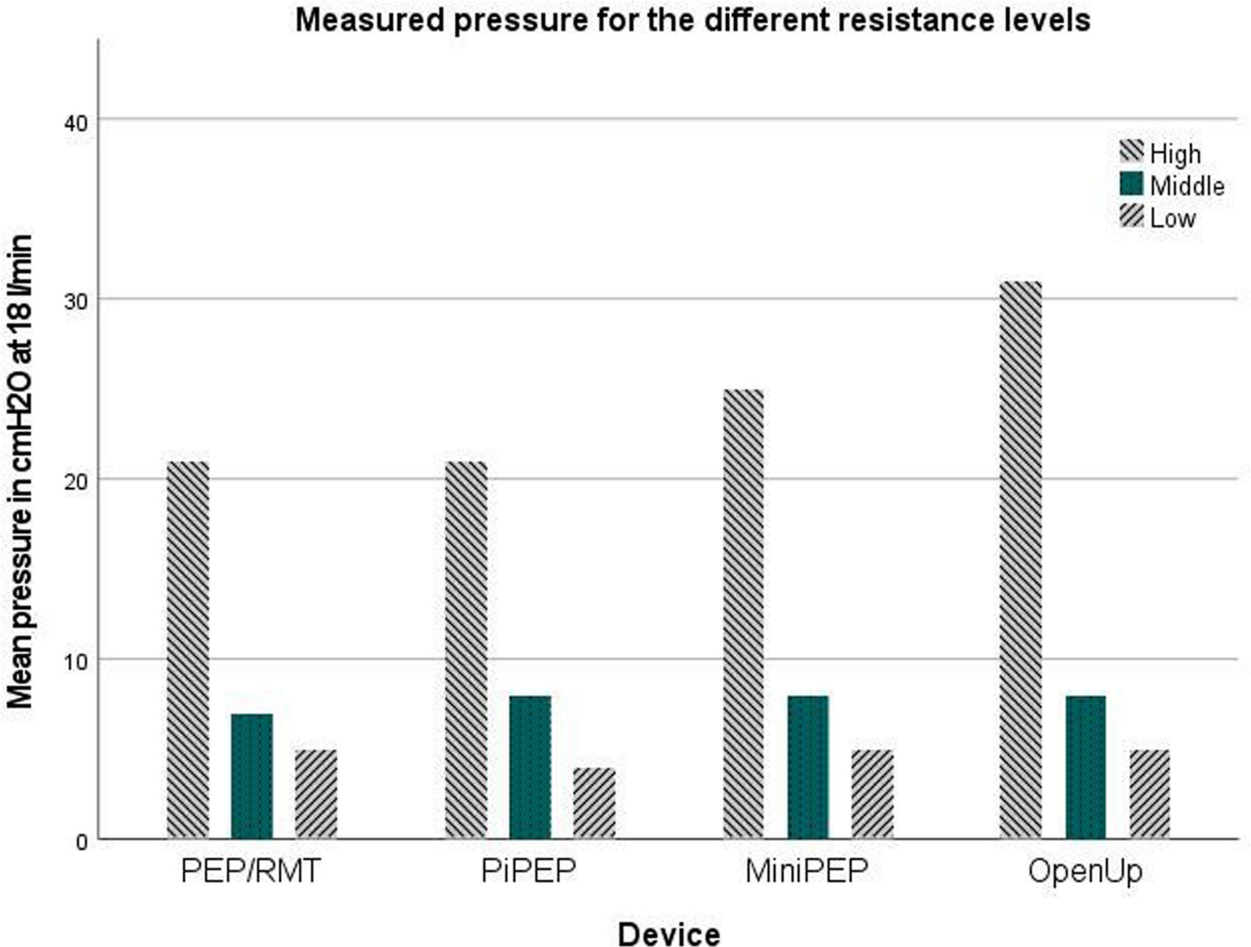
Pressures measured at a flow of 18 L/min in OpenUp Flow, compared with the competitors PEP/RMT system, PiPEP, and MiniPEP. High resistance was compared with 2.5 mm resistors. Middle resistance was compared with 3.5 mm resistors. The low resistance was compared with 4.0 mm resistors.

The study was conducted at the School of Health and Welfare, University of Jönköping, Sweden. Tests were performed at standardized flow rates of 10 and 18 L/min, with each resistance level assessed for 10 s at both rates and repeated three times, and the mean value was used in the evaluation. The flow rates of 10 and 18 L/min, respectively, have been used during previous testing of PEP devices [5]. The flow rates of 10 and 18 L/min, respectively, were chosen to compare OpenUp Flow with other existing PEP devices on the market that previously have been tested [5] (Figures 2 and 3).

The gas system at the test site and a gas mounted flowmeter RTM 3 (Technologie Medicale Noisy le Sec, France) was used to achieve a constant airflow. A tubing (Mediplast AB, Malmö, Sweden) was moulded into the OpenUp Flow device to allow the flow into the device, and another tube was created as an outlet to measure the pressure with a pressure gauge (Welch Allyn, New York, USA) (Data S2).

### 2.1 Statistics

Data are presented as mean and standard deviation. The results in pressure when testing the different hole‐blocking combinations of OpenUp Flow were analysed. Pairwise comparisons between the measured pressures during flows of 10 and 18 L/min were analysed with independent‐samples Kruskal–Wallis test. Data obtained at 10 and 18 L/min were analysed separately. Statistically significant differences were set at a *p* value of < 0.05. SPSS version 28.0.1.1 was used for the statistical analyses.

## 3 Results

A range of 3–31 cmH_2_O was generated with flows of 10 and 18 L/min. During a flow of 10 L/min, pressures ranged from 3 cmH_2_O (no blocked opening) to 11 cmH_2_O (two blocked openings). During tests with a flow of 18 L/min: pressures ranged from 5 cmH_2_O (no blocked openings) to 31 cmH_2_O (two blocked openings) (Table 1).

**TABLE 1:** Measured mean pressure in cmH_2_O at the three resistance levels in OpenUp Flow (Parx AB, Saltsjöbaden, Sweden) during standardized flows in litres/min.

Resistance	10 L/min	18 L/min
High (two blocked openings) *	11 (0)	31 (0.5)
Medium (one blocked opening)	4 (0)	8 (0)
Low (no blocked openings) *	3 (0)	5 (0)

*Note:* Measured mean pressure in cmH_2_O (SD).

* The difference between high and low resistance were significant at 10 and 18 L/min (*p* = 0.018 and *p* = 0.020, respectively).

The overall difference between the pressure levels was significant, at both 10 L/min (*p* = 0.018) and at 18 L/min (*p* = 0.02). At 10 L/min, blocking of two holes increased pressure significantly compared with no blocked hole (*p* = 0.005), but not compared with one blocked hole (*p* = 0.157). At 18 L/min, blocking of two holes increased pressure significantly compared with no blocked hole (*p* = 0.005), but not compared with one blocked hole (*p* = 0.163).

## 4 Discussion

The study findings reveal an expected relationship between higher resistance levels and the generated pressure in the tested PEP device. At both 10 and 18 L/min flow rates, the pressure output varied substantially across different resistance settings, especially between blocking of two holes compared to blocking of one or no hole. This variability is caused by to the increasing turbulent flow at higher flows.

To compare OpenUp Flow with existing PEP devices on the market previous published data of tested devices have been used here [5] (Figures 2 and 3). The following PEP devices with resistors have previously been tested using flows of 10 and 18 L/min; PEP/RMT (Mediplast AB, Malmö, Sweden), PiPEP Breathing Exerciser (Koo Medical Equipment, Hong Kong, China) and Mini‐PEP (Philips with resistors from Rûsch, Duluth, Georgia). There was no significant difference regarding pressure regulation at 10 or 18 L/min (*p* > 0.05), between OpenUp Flow and the comparable reported data from the PEP/RMT system, PiPEP, and MiniPEP [5]. At 10 L/min, the OpenUp Flow high resistance (two blocked holes) corresponds to the 2.0 mm resistors. The medium OpenUp Flow resistance (one blocked hole) corresponds to 3.0 mm resistors. The low resistance (no blocked hole) corresponds to 3.5 mm resistors. At 18 L/min, the high resistance (two blocked holes) in OpenUp Flow corresponds to the 2.5 mm resistors. The medium (one blocked hole) OpenUp Flow resistance corresponds to the 3.5 mm resistors. The low resistance (no blocked hole) corresponds to the 4.0 mm resistors. There is a difference between all the compared resistors mentioned in this paper that do not make them interchangeable but comparable [5]. This study demonstrates that the pressure generated during standardized tests of OpenUp Flow has comparable properties, but not interchangeable, to that of other resistors available in the market. It is important to consider the technical details when prescribing breathing exercises and PEP devices, as one‐size‐fits‐all solutions are not suitable for everyone [6]. A clinical expiratory target pressure 10–15 cmH_2_O during postoperative physical therapy is common [4]. OpenUp Flow consistently generates pressure well within this recommended pressure range.

OpenUp Flow does have limitations: It does not differentiate between inspiration and expiration like advanced systems do. Patient cooperation is needed for nasal or oral inspiration, proper lip seal and device removal, which may be challenging. It lacks expiratory pressure measurement capability, is incompatible with masks and lacks provision for connecting to supplemental oxygen.

One limitation of this study is its laboratory setting, which limits assessing real‐world clinical use and patient input. The standardized flow rates used for evaluation in this trial do not entirely correspond to COPD patients but serves well for comparison between devices and define its properties [7, 8]. The selection of flow rates of 10 and 18 L/min was based by previous studies on PEP‐therapy [5].

## 5 Conclusion

This trial of the pressure regulation capabilities of OpenUp Flow demonstrates a pressure range of 3–31 cmH_2_O achievable with flow rates of 10 and 18 L/min. At 10 L/min, pressures ranged from 3 to 11 cmH_2_O, while at 18 L/min, pressures ranged from 5 to 31 cmH_2_O. Overall, there was a significant difference in pressure among resistance levels at both flow rates, showing that blocking two holes significantly increased pressure compared to no blocked hole.

## Author Contributions

Data collection: B.S. and P.W. Study design: P.W. and B.S. Analysis of data: P.W. Manuscript preparation: P.W. and E.W. Review of manuscript: All authors reviewed the manuscript and approved the final submitted version.

## Conflicts of Interest

Pär Wennberg is a partner of Parx AB who have developed OpenUp Flow. The rest of the authors declare no conflicts of interest.

## Supporting information


**Data S1.** Description of the placement of the openings. Opening area of all openings are 5 mm^2^. All measures are in mm.

**Data S2.** Test setup. Wall mounted gas outlet, flowmeter, OpenUp Flow device and pressure gauge.

## Data Availability

The data that support the findings of this study are available from the corresponding author upon reasonable request.
