# Supplementary figures and images for: Pressure Properties of a New Positive Expiratory Pressure Device—OpenUp Flow a Three‐in‐One Solution
Source: Clin Respir J. 2025 May 13;19(5):e70084. doi: 10.1111/crj.70084 (PMC12070251; doi:10.1111/crj.70084)

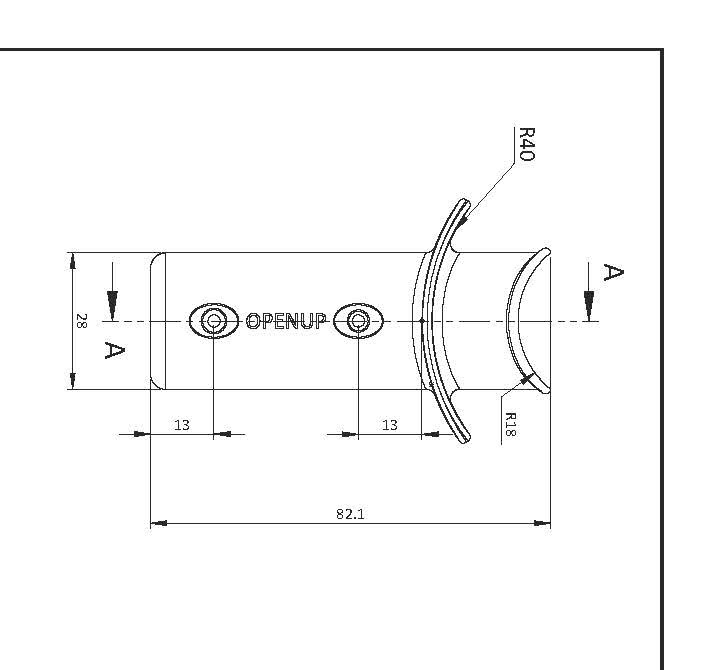

Supplement: Supplementary file 1 — Data S1. Description of the placement of the openings. Opening area of all openings are 5 mm2. All measures are in mm. [file CRJ-19-e70084-s002.jpg]

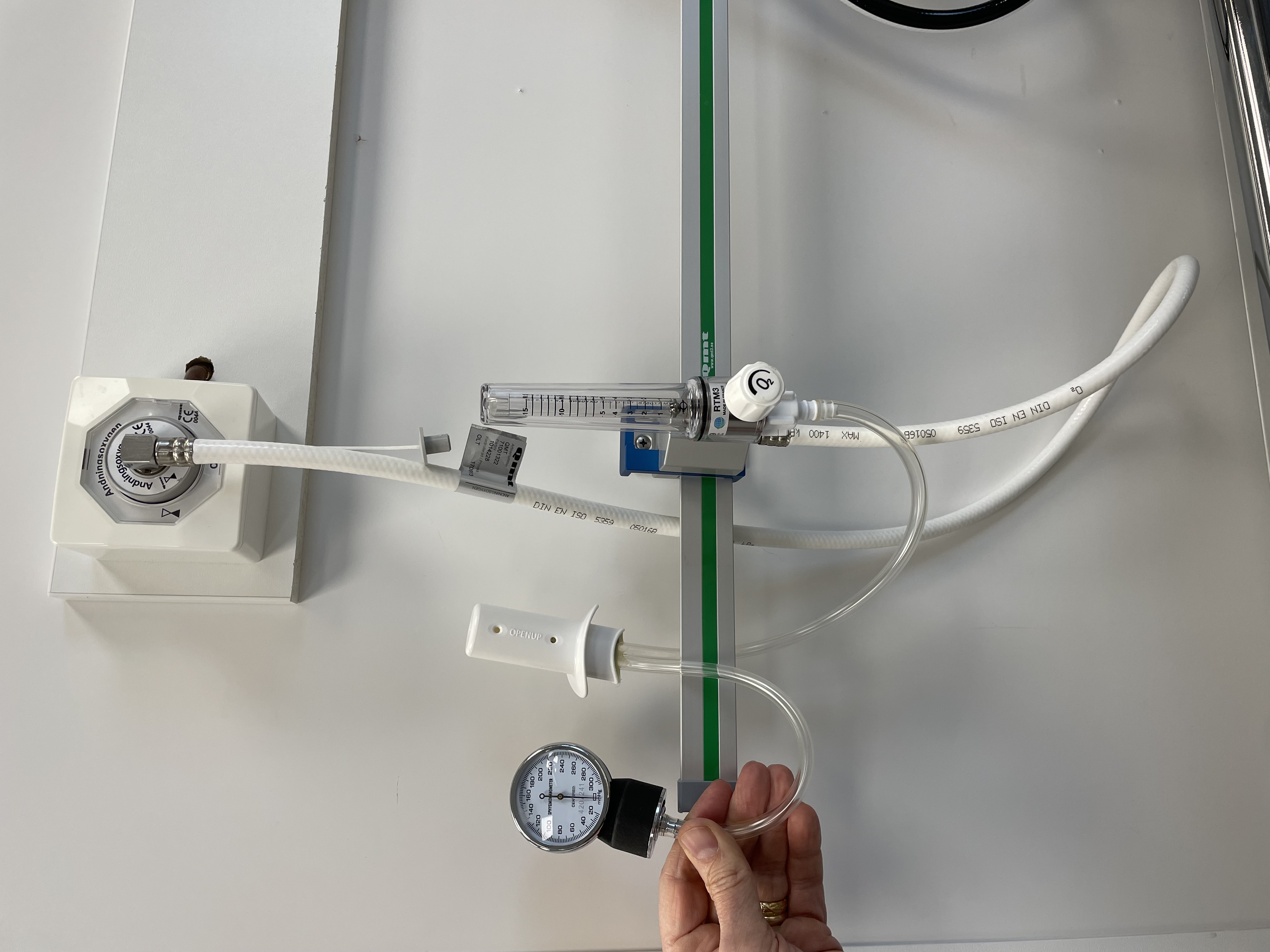

Supplement: Supplementary file 2 — Data S2. Test setup. Wall mounted gas outlet, flowmeter, OpenUp Flow device and pressure gauge. [file CRJ-19-e70084-s001.jpg]
